# Electrochemically Activated CNT Sheet as a Cathode for Zn-CO_2_ Batteries

**DOI:** 10.3390/ijms232012602

**Published:** 2022-10-20

**Authors:** Daniel Rui Chen, Megha Chitranshi, Vesselin Shanov, Mark Schulz

**Affiliations:** 1 Department of Mechanical and Materials Engineering, University of Cincinnati, Cincinnati, OH 45221, USA; 2 Department of Electrical Engineering and Computer Science, University of Cincinnati, Cincinnati, OH 45221, USA; 3 Department of Chemical and Environmental Engineering, University of Cincinnati, Cincinnati, OH 45221, USA

**Keywords:** CNT/Cu composite, CO_2_ reduction, Zn-CO_2_ battery

## Abstract

High demand for electrochemical storage devices is increasing the need for high-performance batteries. A Zn-CO_2_ battery offers a promising solution for CO_2_ reduction as well as energy storage applications. For this study, a Zn-CO_2_ battery was fabricated using a Carbon Nanotube (CNT) sheet as a cathode and a Zn plate as an anode. The electrochemical activation technique was used to increase the surface area of the CNT electrode by roughly 4.5 times. Copper (Cu) as a catalyst was then deposited onto the activated CNT electrode using electrodeposition method and different Cu loadings were investigated to optimize CO_2_ reduction. The final assembled Zn-CO_2_ battery has a 1.6 V output voltage at a current density of 0.063 mA/cm^2^, which is higher than most devices reported in the literature. This study demonstrates the importance of activation process which enabled more catalyst loading on the cathode resulted in additional active sites for electroreduction process. This paper presents the activated CNT sheet as a promising cathode material for Zn-CO_2_ batteries.

## 1. Introduction

The Metal-CO_2_ battery has attracted attention due to its unique approach to utilize CO_2_ economically and efficiently. The discharging process of the battery is based on the mechanism that naturally occurred the oxidation process from the metal anode provide electron to reduce CO_2_ to various hydrocarbon byproducts, electrons can pass through an external circuit to the cathode and generate electricity [1]. Although metal-CO_2_ battery research is still at a very early stage, it is considered to be a promising solution to capture and recycle CO_2_. This technology is intended to address two major global issues that are currently faced by humanity: the energy crisis and climate change caused by the rapid rise of CO_2_ levels.

Various successful metal-CO_2_ batteries have been demonstrated in recent years including Li-CO_2_ [2,3,4,5], Al-CO_2_ [6]_,_ Na-CO_2_ [7], and others. The most studied battery is Li-CO_2_ battery due to its high theoretical energy density of 1876 Wh Kg^−1^ [2], compared to other available batteries. However, these batteries have challenges including, use of high active metal anodes, solid discharge products, and cost limits the practical environmentally friendly applications [8,9,10]. Zn-CO_2_ provides a promising alternative due to its low toxicity, safety, cost, low active metal anodes, and is environmentally friendly [4,8,9,11,12].

Reutilizing CO_2_ to produce other high-value chemicals is deemed to be a more practical route. Electrochemical reduction of CO_2_ (ERC) has many advantages, which include producing high economic value products such as methanol, ethylene, and formic acid, operating at room temperature and atmospheric pressure, and the process can be powered using renewable sources of energy [13].

However, ERC has also been associated with many constraints and disadvantages. These shortcomings are demonstrated in the following three points: (i). ERC has suffered from high electrode overpotential and low resultant current density [14,15]; (ii). The hydrogen evolution reaction competes with the ERC process at the high potential in the aqueous phase [16]; (iii). Product selectivity and low efficiency often plague the ERC process [15]. Using electrocatalysts has been suggested to lower the potential and increase the current density, and to improve the overall efficiency of the ERC process. Metal and semiconducting metals, such as Cu, Fe, Sn, Au, Pt, etc., are commonly used as cathode materials to reduce CO_2_ electrocatalytically [17,18]. 

Among various metal catalysts, Cu has been considered the most appropriate candidate for the ERC process. In an aqueous phase, Cu can electrocatalytically reduce CO_2_ to hydrocarbons with relatively low current density (around 5–10 mA cm^−2^) and high current efficiency (i.e., >69% at 0 °C) [19]. Furthermore, the selectivity of the products can be controlled by the atomic structure of Cu, for instance, a Cu (100) electrode with an optimal density of steps has shown higher selectivity for C-C coupling than other planar surfaces [20,21]; production of oxygenates is enhanced in the crystal with the plane of (100) and (110) [20,22]; recent DFT results also show that Cu (211) yields higher magnitude activity than other planes (100) and (111) for CO reduction to CH_4_ [23]. 

CNT materials have been known for their great electrical and mechanical properties [24,25], chemical inertness, and electrocatalytic stability [26], as well as high surface area [27]. All these advantages make CNT a good substrate material for Cu deposition. By utilizing the porous surface morphology of the CNT sheet, Cu materials can penetrate and distribute more evenly inside the nanotubes. The inherent mechanical property of CNT also provides CNT-Cu composite materials with a great degree of flexibility. To ensure the formation of micro-sized Cu particles, a slow-paced organic deposition method was used to deposit Cu on the CNT sheet. Two Zn-CO_2_ batteries were investigated using two tubular cathodes prepared from directly grown CNTs on copper and nickel mesh [28]. These batteries demonstrated excellent stability, high input voltage, and discharge performance. Another Zn-CO_2_ was fabricated using hollow CNT fiber exhibited high selectivity and efficiency [10]. The battery showed energy density of 288.3 Wh Kg^−1^ and was stable up to 8 days.

This paper discusses the use of CNT sheet as a cathode material for Zn-CO_2_ battery. The CNT sheet was first acid-treated to make it hydrophilic and then electrochemical activation was performed on the acid-treated CNT sheet to increase the surface area for Cu deposition. The organic deposition method was used to deposit the Cu on the activated CNT sheet, different Cu depositions were achieved by varying the deposition time of the process. Optimum deposition time in terms of CO_2_ reduction efficiency was studied. The aim of this research is to use activation process to increase the surface area of the electrode for more catalyst deposition. The additional area will provide more activation sites for electrochemical reduction activity and improve the performance of the device. The performance of Cu deposited CNT was then investigated and compared with non-activated CNT cathode electrode.

## 2. Results & Discussion

### 2.1. CNT Activation and Cu Deposition

**Figure 1:**
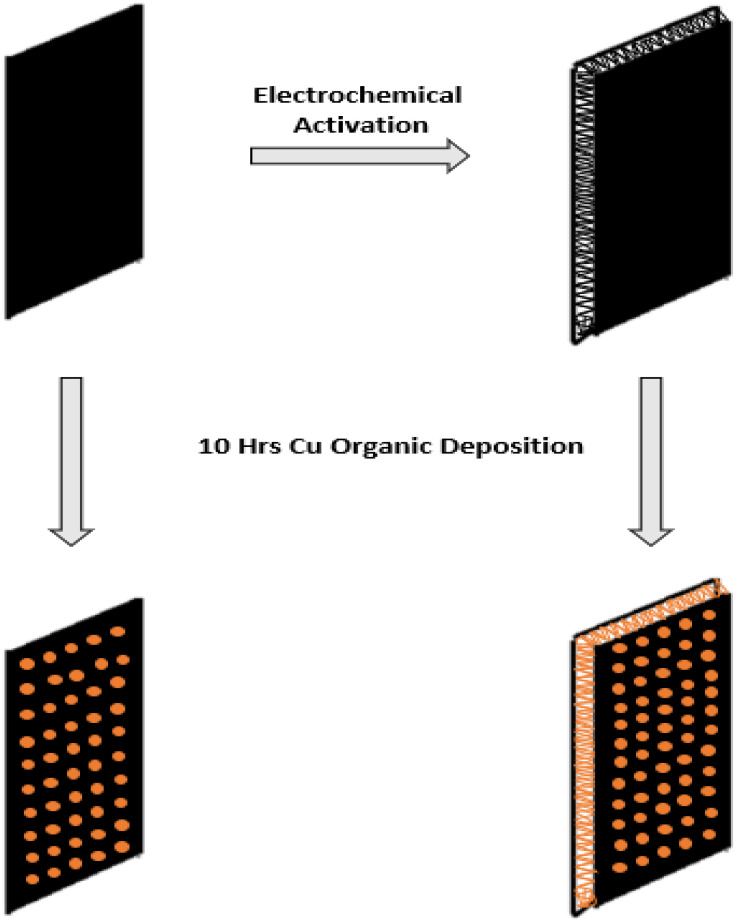
The illustration of process steps used for preparing CNT samples for analysis.

During the final stage of the FCCVD process for CNT synthesis, the CNT sheet has lost a significant amount of surface area due to the densification process. Thus, electrochemical activation has been used as a practical approach to recover some of the losses. The activation is explained in detail in our previous paper [29]. Before the activation, CNTs sheets were acid-treated to increase their hydrophilicity The hydrophobicity of as-synthesized CNT sheet has prevented electrolytes from the wetting the electrode, which is mainly due to the nonpolar aromatic molecular structure. Acid treatment was used to functionalize the CNT sheet and promote the wetting [30]. Additionally, to not bring external chemical elements into the electrochemical cell, NaCl was used as the electrolyte to activate the CNT sheet. The duration of activation was limited to less than 10 min to prevent CNT structural damage caused by excessive activation. During the activation process, electrolysis, and formation of H_2_ caused expansion and delamination of the CNT sheet. After 10 min activation, the activated CNT sheet exhibited sponge-like and multi-layer morphology, which was able to increase the inner surface area for the deposition of Cu. The illustration of activation and deposition process used for CNT sheet samples for CO_2_ reduction and battery part is shown in Figure 1.

During the Cu deposition, organic electrolytes (*Cu (CH*_3_*COO)*_2_ *& CH*_3_*CN*) were used for deposition. Organic deposition has been used as a Cu seeding method in some publications, it has helped to achieve a uniform and less congregated Cu distribution on CNT substrate due to its low Faradaic efficiencies and slow deposition rate [31]. In comparison to an aqueous electrolyte deposition method (*CuSO*_4_), organic deposition can infiltrate into the activated area and provide more balanced internal and surface deposition [31]. In this research, organic deposition was used to deposit Cu onto activated CNT sheet to maximize its surface area.

Cu deposition was achieved by electrochemical deposition of Cu onto a CNT electrode with a constant voltage by using the organic deposition method. The organic deposition method (OD) has been used to promote Cu deposition within CNT wires and control surface deposition to obtain a uniform and continuous Cu matrix. OD is the initial seeding process that deposit nano-sized Cu particle, under the following heat treatment, these nanosized Cu particles can agglomerate into micro-sized Cu particles, which catalyze CO_2_ reduction [31,32]. During the CV measurement, the three electrode method was used to measure the cyclic voltammetry at different scan rates (10, 25, 50, 100, and 150 mV/s), the CV curves were presented in Figures S1A–D and S2 (Sections S1 and S2) to study the electrochemical activity of various samples, according to which there is no redox peak for the acid-treated sample, whereas redox peaks are visible for the 2, 5, 10, 20 h for the Cu deposited sample. The onset of the reduction peak is an indication of the occurrence of the CO_2_ reduction reaction. Due to the absence of Cu catalyst, a redox reaction is unlikely to have occurred on the acid-treated CNT sample.

Additionally, the relationship between current density and potential is shown in Figure S2, as it indicated that current density increases with the increase in potential. However, the acid-treated CNT sample shows the lowest negative current density increase compared with other samples. The 10 h Cu deposited sample showed the highest negative current density increase especially below −1 V. Due to its great electrochemical activity at a negative potential, 10 h was considered the optimal OD time frame to provide most electrochemically active CNT sample for CO_2_ reduction.

### 2.2. The Surface Area Comparison

The electrochemical active surface area of acid-treated CNT as well as CNT after activation was estimated based on the double-layer capacitance (C_dl_) by using a cyclic voltammetry (CV) graph. According to the classic capacitance equation for double-layer capacitance: $C_{dl}=\varepsilon_{0}*A/\;d$, where *A* is the surface area and d is the distance between layers [33], the surface area of the electrode is proportional to the capacitance value.

**Figure 2:**
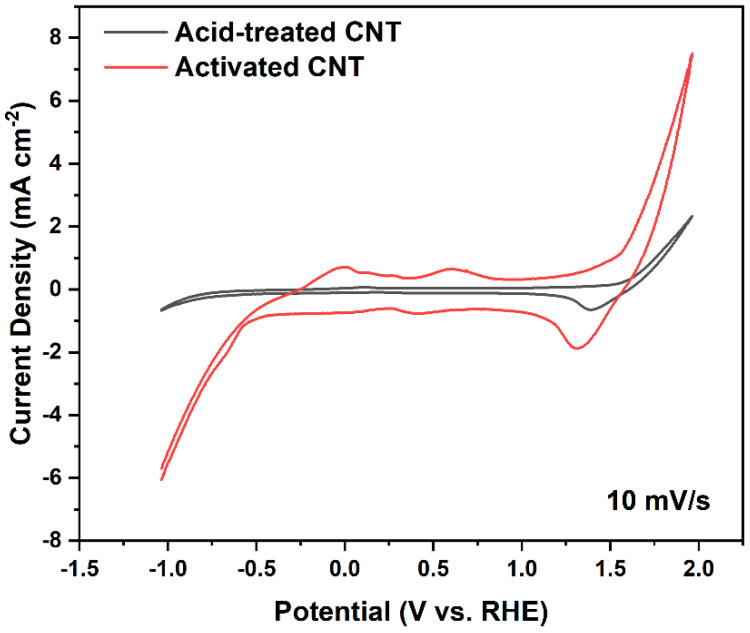
CV curve of acid-treated and activated CNT.

During the measurement, CV was taken between −1.0 and 2.0 V vs. RHE at scan rates of 10 mV/s and the electrolyte is 1 M NaCl saturated with CO_2_. According to the CV graph in Figure 2, the capacitance of the sample was estimated using $C_{dl}=\int Idv/v*m*\Delta V$, where ∫Idv is the integrated area under the CV curve, v is the scan rate, m is the weight of the electrode sample, and ΔV is the voltage window. Because v, m, and ΔV are the same for the acid-treated and activated samples, the surface area ratio between activated CNT and acid-treated CNT is equivalent to the ratio of the integrated area of their respective CV curve. Based on the calculation, ∫Idv of the activated CNT curve is around 4.5 times larger than the acid-treated CNT curve, which suggested a large increase in the electrochemical active area and higher surface roughness.

### 2.3. Structural Analysis

**Figure 3:**
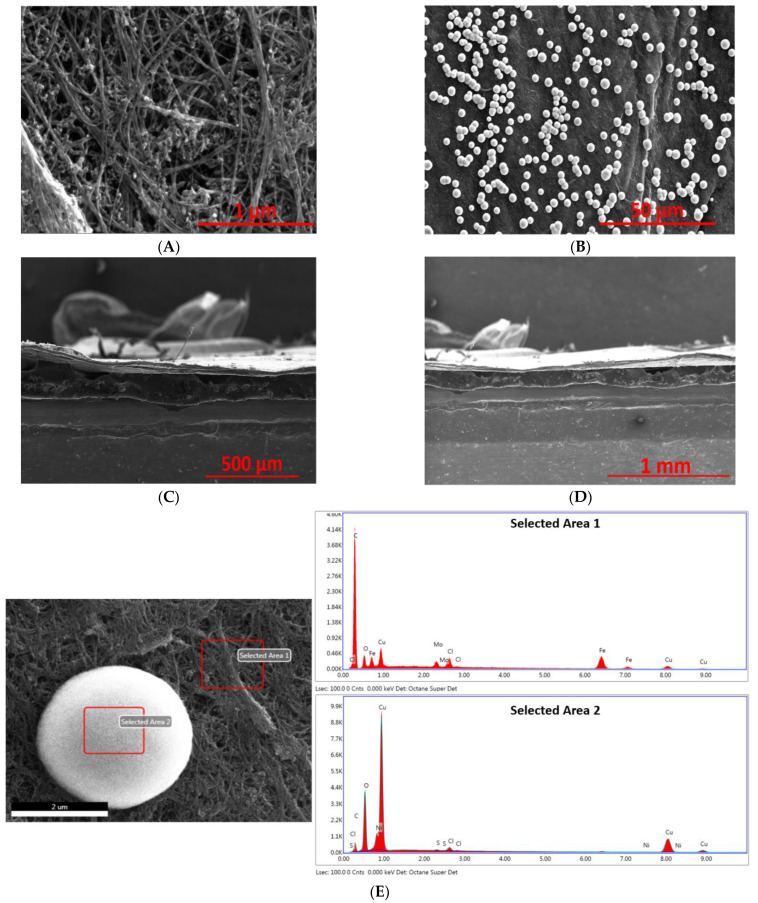
SEM images of acid-treated CNT with Cu organic deposition: surface morphology (**A**,**B**), as well as cross-sectional images (**C**,**D**), and EDX spectrum of the selected area (**E**).

**Figure 4:**
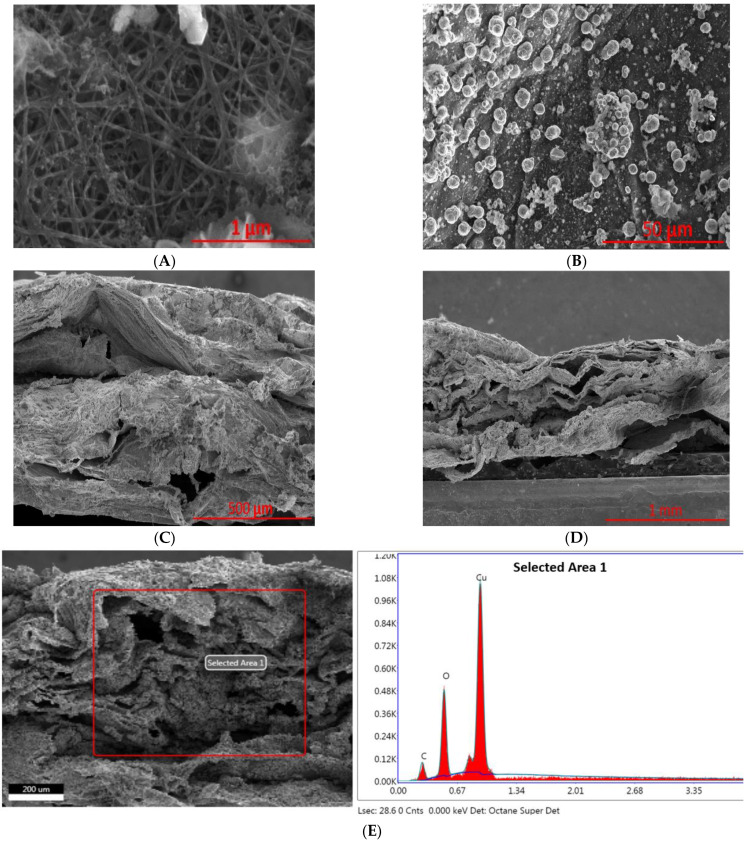
SEM images of activated CNT with Cu organic deposition: surface morphology (**A**,**B**), as well as cross-sectional images (**C**,**D**), and EDX spectrum from cross-sectional area of the activated sample (**E**).

The SEM images of acid-treated and activated CNT with Cu deposition with different magnification as well as their EDX graph are shown in Figure 3 and Figure 4, respectively. After organic deposition, Cu precipitant was first formed around CNT strands and deposited onto the surface of CNT sheet (Figure 3A). After the heat treatment, these precipitants started to coalescence and formed large size spherical particles, which were uniformly distributed on the CNT surface as shown in Figure 3B, while the cross-section is non-activated (Figure 3C,D) and its compact structure prevents further infiltration of Cu. Additionally, EDX was used to verify the presence of Cu (Figure 3E). The presence of O indicates that Cu particles are partially oxidized, a small percentage of impurities such as Fe, S are from the catalyst source (Ferrocene) and growth promoter (Thiophene), respectively, used in the nanotube synthesis process. The elements of Mo and Cl are possibly from the ceramic tube used in the synthesis reactor.

In terms of the activated CNT sheet, due to the nature of the electrochemical activation process, which generates a copious amount of H_2_, H_2_ is not only able to delaminate the CNT sheet, but surface roughness has also been increased due to the bulge formation in the CNT sheet from H_2_ generation. The distance between CNT strands appears to be winder that of non-activated ones (Figure 4A), the rough surface also gives rise to the rough Cu surface morphologies (Figure 4B). Past literature has shown that surface roughness has a direct relationship with activity and product selectivity of CO_2_ reduction [34,35]. There are more under-coordinated sites are present in the roughened surface that showed more adsorption energies compared to highly coordinated sites. Cross-section SEM images showed the multilayer and delaminated structure, which provide ample space for Cu deposition. An EDX spectrum of the cross-section indicates that Cu has a large presence, and the presence of O indicates Cu is partially oxidized.

### 2.4. Linear Sweep Voltammetry Graph and Potentiostatic Curve

**Figure 5:**
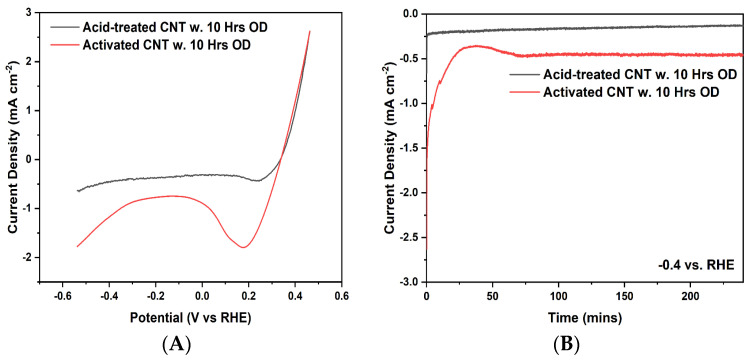
LSV curve (**A**) and potentiostatic curve (**B**) of acid-treated CNT with 10 Hrs Cu organic deposition and activated CNT with 10 Hrs organic deposition.

A linear sweep voltammetry (LSV) graph was used to estimate the electrochemical reduction potential of the electrodes. Enhanced current density at increased negative potential suggests high electrocatalytic activity of activated CNT/Cu for CO_2_ reduction. The comparison was made between Cu deposited acid-treated CNT and Cu deposited activated CNT, before the LSV measurement, NaCl electrolyte was saturated with CO_2_, and CO_2_ gas was continuously pumped into NaCl electrolyte during the experiment. The range of sweeping potential was kept between 0.4 and −0.6 vs. RHE, current density was normalized by the geometric area of the CNT sample. It can be seen from Figure 5A that, for the Cu deposited activated CNT sample, a rapid shift in current and increased current density indicate an intensified reduction reaction. The reduction current density initially decreased and increased with further increase of reduction potential. However, in terms of Cu deposited only in the acid-treated sample, the current density appeared to be stabilized at around 0.5 mA/cm^2^ after the initial rapid decline, which indicates a minor occurrence of the electrochemical reduction reaction.

Potentiostatic testing was carried out and results are shown in Figure 5B. A constant voltage −0.4 vs. RHE was applied and the current density variation was measured during the 4 h running. Cu deposited activated CNT experienced initial decline and later became stabilized around 0.5 mA/cm^2^, while Cu deposited only acid-treated CNT was kept constant around 0.25 mA/cm^2^, which indicates that activated CNT provides extra space for Cu deposition to increase the activation sites, additionally rough surface morphology of Cu particles on the CNT sheet enhances the electrochemical activities.

### 2.5. Zn-CO_2_ Battery Performance

**Figure 6:**
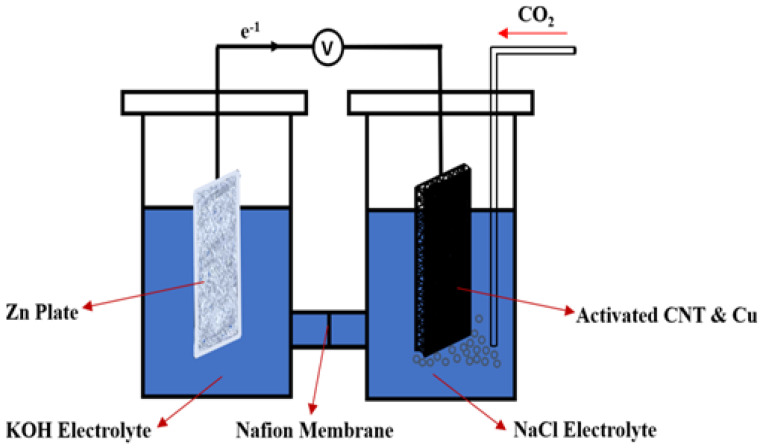
Schematic diagram of the Zn-CO_2_ electrochemical cell.

The Zn-CO_2_ metal battery was constructed in the H-cell, which is schematically shown in Figure 6. Cu deposited activated CNT was used as the cathode material in the cathodic compartment, while the Zn plate was used as an anode in the anodic compartment. A Nafion membrane was used to stabilize the pH values of anolyte and catholyte. After NaCl was saturated with CO_2_, the gas was constantly bubbling into the electrolyte during the experiment. During the discharge process, the Zn plate can react with KOH to form Zn (OH)_2_ and release electrons, at the same time, CO_2_ reduction occurred on the Cu deposited CNT electrode, and Cu catalyzes to promote the CO_2_ conversion. 

The possible reaction occurred on both electrodes are shown below:

***Cathode***: 2CO_2_ + 8H^+^ + 8e^−1^ ↔ CH_3_COOH +2H_2_O (1M NaCl, sat. CO_2_) (1)

***Anode***: 4Zn + 8OH^−^– 8e^−1^ ↔ 4Zn (OH)_2_ (1M KOH) (2)

***Overall reaction***: 4Zn + 2CO_2_ + 8OH^−^ +8H^+^ ↔ 4Zn (OH)_2_ + CH_3_COOH +2H_2_O (3)

**Figure 7:**
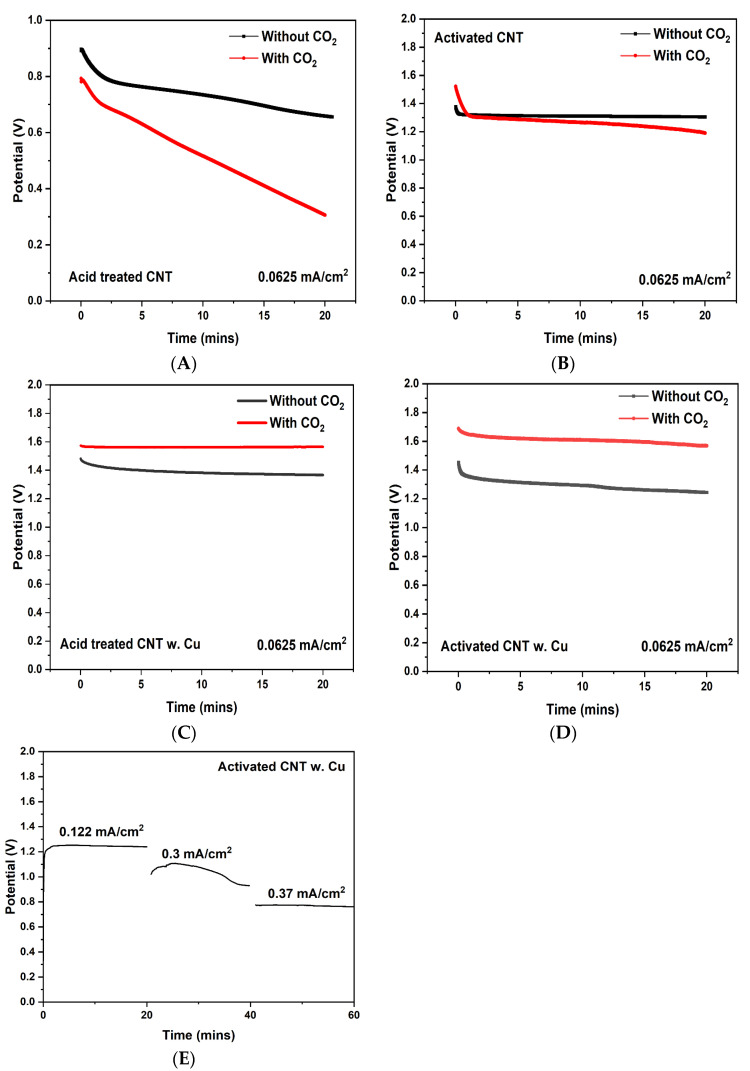
Discharging curve of acid-treated CNT for 20 min (**A**), activated CNT (**B**), Cu-deposited acid-treated CNT (**C**), Cu deposited activated CNT (**D**), discharging curve of Cu deposited activated CNT at different current densities (**E**).

The potential stability of Zn-CO_2_ in terms of discharging time before and after the saturation of CO_2_ was measured_._ According to Figure 7A, the acid-treated CNT showed the lowest potential output and was followed by a sharp potential drop, while inactivated CNT. In Figure 7B, before the saturation of CO_2_, the output potential of a cell has been stabilized around 1.3 V, after the saturation of CO_2_, the potential has seen a steady drop, which is likely due to the increased internal resistance caused by CO_2_ saturated electrolyte. Figure 7C,D, both demonstrate the potential to increase due to the introduction of CO_2_, furthermore, Cu deposited activated-CNT shows the highest potential increase of 0.4 V after CO_2_ saturation, while Cu deposited acid treated-CNT has a 0.2 V increase after CO_2_ saturation. During 20 min discharging process, the discharge current density was kept at 0.0625 mA/cm^2^. The potential change at different current densities was also measured and presented in Figure 7E, the potential was reduced from 1.2 V at a current density of 0.122 mA/cm^2^ to 0.8 V at a current density of 0.37 mA/cm^2^. In comparison, when NaHCO_3_ was used as electrolyte on both side of the cell, due to the low reactivity between Zn and NaHCO_3_, output potential show negligible improvement when Cu-CNT was cathode (Supplementary Materials Figure S3 in Sections S3). 

**Figure 8:**
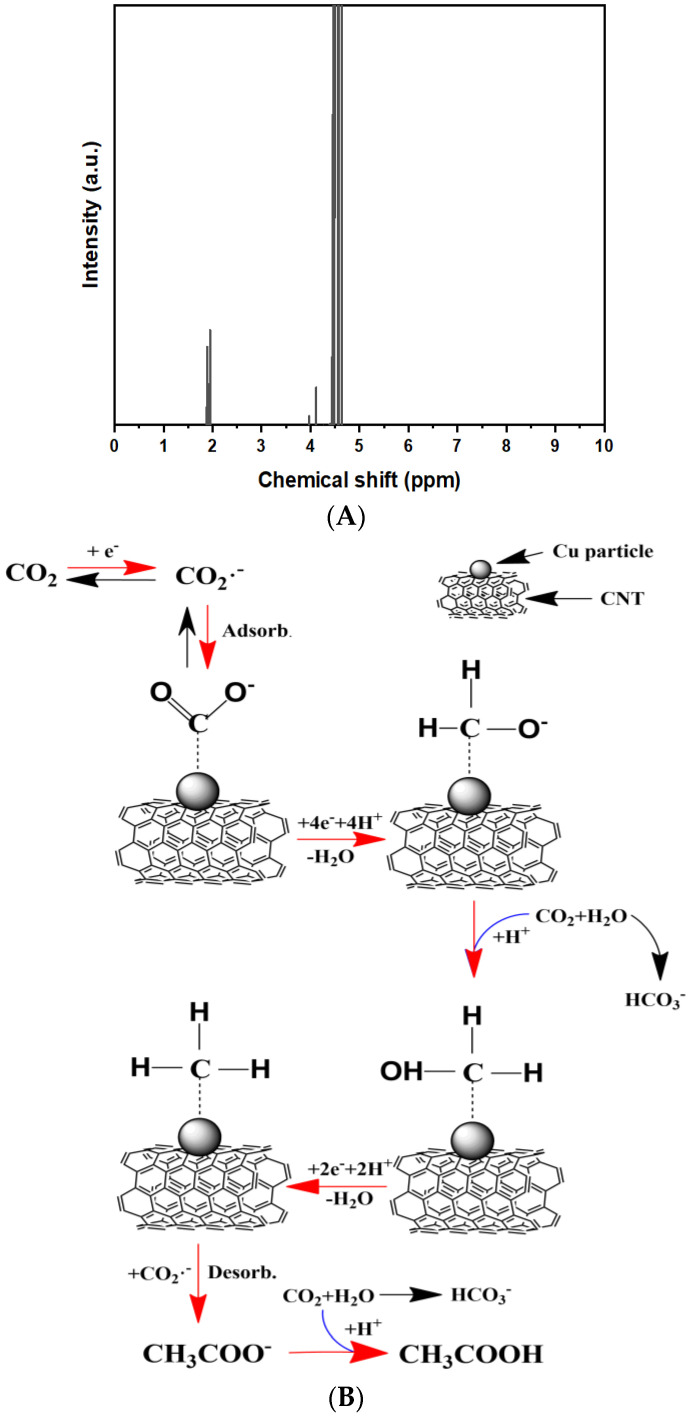
1H NMR graph of the by-product electrolyte (**A**) and acetic acid by possible product pathway from CO_2_ reduction (**B**).

The formation of acetic acid (CH_3_COOH) in the electrolyte has been verified by Nuclear Magnetic Resonance (NMR) (Figure 8A). NMR is used to determine the molecular structure at the atomic level, in which all nuclei have multiple spins and are electrically charged. Inside of NMR magnetic field, radio wave excites the nuclei from lower to higher energy lever in a single step and generates nuclear magnetic resonance, which can be detected by sensitive radio receiver. The resonance frequency gives access to the detailed information of the electronic structure and functional group of a molecule/chemical compounds [36,37]. 

According to various literature, pathways towards C2+ products are considered to be very complex and generates debate among researchers performing theoretical studies. Compared with C1 products, C2+ hydrocarbon by-products contain two or more carbon atoms and have higher energy densities, although most theoretically proposed mechanisms are based on density functional theory (DFT) simulations, however, the consensus is by transferring a concerted proton-electron (H^+^/e^−^) from the electrolyte to adsorbed species, which in this case is Cu surface, a CO_2_ molecule can reduce to carboxyl intermediate *COOH and then to *CO and water molecule, further reaction include protonation of *CO to *CH3O and following coupling with CO_2_·^−^ to form to acetate [38], which is illustrated in Figure 8B due to the complicated nature of the reaction as well as this could well be one of the possible routes to form acetic acid, further studies are needed to fully confirm. The Zn-CO_2_ battery has a theoretical electromotive force of 4.557 V and a theoretical energy density of 915.75 Wh Kg^−1^ (Supplementary Materials Section S4). The comparison between this work and other works can be found in Table S1 in Section S5 which proves that this Zn-CO_2_ battery has a high open voltage of 1.6 V compared to other batteries reported in the literature. 

## 3. Materials and Methods

### 3.1. The Synthesis and Activation of CNT Sheet

The CNT sheet used in this experiment was synthesized from Floating Catalyst Chemical Vapor Deposition (FCCVD) method. The FC-CVD process is a one-step continuous process and can produce industrial-scale nanotube sheet. The process is substrate-free and provides flexibility to tune the process and customize the material according to a specific application. In this process, we introduced a feedstock at the inlet of the reactor. The feedstock is composed of methanol, n-Hexane, ferrocene, and thiophene. The CNT sock (web of nanotubes) forms in the high-temperature synthesis zone of the reactor and is collected at the outlet of the reactor onto a rotating drum. A detailed description of the synthesis process tuning, process analysis, and characterization techniques can be found in our previously published research articles [39,40,41,42,43,43,44,45,46]. The as-synthesized CNT sheet materials are cleaned and expanded through an acid treatment and activation process. The acid treatment follows the steps of immersing the sample inside a mixture of 3:1 ratio of H_2_SO_4_ (*Fisher Chemical*) and HNO_3_ (*Fisher Chemical*) followed by heat reflux (90 °C) for 2–3 h.

For activation of the CNT sheet [29], a three-electrode method was employed using an electrochemical workstation (*Gamry, Interface 1000, Warminster, PA, USA*). A rectangular CNT sheet (*34 mm × 48 mm*) served as the working electrode. Ag/AgCl and graphite rods were used as the reference and counter electrodes respectively. A 0.3 M NaCl solution was used as the electrolyte. A cyclic voltammetry (CV) sweep was conducted at a scan rate of 50 mV/s, with an applied voltage between −2 V and +2 V, to chemically activate which increases the surface area of the CNT sheet. Typically, 10–15 scan cycles were considered as the optimal number of cycles to fully activate the CNT without causing structural damage to the CNT sheet. The relationship between number of activation cycles and optimal electrochemical performance is given in [29]. 

### 3.2. Organic Deposition of Copper on Activated CNT

The Cu electrodeposition was carried out on a workstation equipped with a power supply (*Circuit Specialists, CSI5003X5, AZ, USA*), a voltage stabilizer (*XY-SK35 CNC buck-boost, Econede, FL, USA*), and a time relay device (*XY-WJ01 Delay Relay Module, Hardware, TKXEC, China*) were used to ensure constant voltage supply and accurate control of the electrodeposition time. The electrodeposition setup consisted of an activated CNT sheet sample as the cathode material that was mounted on a glass slide with a Cu strip attached to the upper edge of the sample. The CNT cathode material and Cu anode material were immersed and separated each other at a distance of ~20 mm in the electrolyte. Anhydrous 0.0075 g copper acetate (*Cu (CH*_3_*COO)*_2_*, Sigma Aldrich, St. Louis, MO, USA*) in 150 mL acetonitrile (*CH*_3_*CN, Sigma Aldrich, St. Louis, MO, USA*) was used as the electrolyte for organic deposition. Deposition times were 2 h, 5 h, 10 h, and 20 h, respectively, and the excess electrolyte after Cu organic deposition was removed from the sample by rinsing in deionized water. The sample was then dried in the air for 1 h. 

### 3.3. Metal-CO_2_ Battery Electrochemical Measurement

Electrochemical activity for CO_2_ reduction experiments was conducted in a three-electrode method using an electrochemical workstation (*Gamry, Interface 1000, Warminster, PA, USA*) and a CV/linear sweep voltammetry (*LSV*) graph was recorded. All electrochemical data was collected vs. the Ag/AgCl reference electrode. The electrolyte used in both compartments was 1 M NaCl solution. The Ag/AgCl electrode and the Platinum electrode were used as the reference and counter electrode. The following equation was used to convert from the Ag/AgCl scale to the reversible hydrogen electrode (RHE) standard:

(4) E (vs. RHE)=E(vs. Ag/AgCl)+0.209V+0.0591V×pH

The pH value of the electrolyte in Equation (1) was measured using a digital pH meter (Digital pH tester pen, VIVUSUN, China).

A full Zn-CO_2_ battery was assembled in an H-type cell separated by a Nafion membrane. A Zn plate (*34 mm × 48 mm*) and Cu deposited CNT were used as anode and cathode respectively. Before the experiment, ultra-pure CO_2_ (*99.99%, Wright Brother, Cincinnati, OH, USA*) was pumped into the cathode compartment for 45 min at a rate of 0.25 sL/min to saturate the electrolyte. The whole system is air-tight to prevent leakage and increase CO_2_ saturation concentration in the electrolyte (*1 M NaCl*). 

### 3.4. Material Characterization

Scanning electron microscopy (SEM) (*FEI SCIOS, 5 kV, Themofisher Scientific, Waltham, MA, USA*) was used to characterize the surface morphology as well as the cross-section of the activated CNT/Cu sample. Nuclear magnetic resonance (*NMR*) (*Bruker NEO 400Billerica, MA, USA*), with Bruker Ascend 9.4 T, 54 mm (narrow) bore, BOSS-336 shim system, equipped with Bruker 5 mm SMART ^15^N- ^31^P and ^19^F observe with ^1^H decoupling and observe, Z-axis PFG, VT range from 150 to +150 °C, was used to analyze the by-product solution from the electrochemical reduction experiment. 

## 4. Conclusions

A Zn-CO_2_ battery with copper deposited CNT as a cathode and a zinc plate as an anode is proposed. The area of the CNT electrode was increased by 4.5 times using the electrochemical activation technique. Different copper loadings were studied for the CO_2_ reduction process, and it was concluded that the ten hours of organic deposition is the optimum loading for our sample. A new approach for using activated CNT as the cathode is presented and the assembled battery showed an open circuit voltage of 1.6 V and a current density of 0.063 mA/cm^2^, which is higher than reported for most device in the literature. Moreover, the oxidized Zn plate is easily replaceable which extends the lifetime of the battery. However, the reduction reaction is still ambiguous and needs a better understanding of the reaction mechanism.

## Data Availability

The data that support the findings of this study are available from the corresponding author upon reasonable request.

## Supplementary Materials

The following supporting information can be downloaded at: https://www.mdpi.com/article/10.3390/ijms232012602/s1.
