# Supplementary material for: Electrochemically Activated CNT Sheet as a Cathode for Zn-CO2 Batteries
Source: Int J Mol Sci. 2022 Oct 20;23(20):12602. doi: 10.3390/ijms232012602 (PMC9604323; doi:10.3390/ijms232012602)
Supplement: Supplementary file 1 [file ijms-23-12602-s001.zip › ijms-1882244-supplementary.pdf]

Supplementary Information

Electrochemically Activated CNT Sheet as a Cathode for Zn-CO_2 batteries

Daniel Rui Chen [1, †], Megha Chitranshi [2, †], Vesselin Shanov [1, 3], and Mark Schulz [2, *]

[1] Department of Mechanical and Materials Engineering, University of Cincinnati, OH 45221, USA

[2] Department of Electrical Engineering and Computer Science, University of Cincinnati, OH 45221, USA

[3] Department of Chemical and Environmental Engineering, University of Cincinnati, OH 45221, USA

† These authors contributed equally to this manuscript.

* Correspondence: schulzmk@ucmail.uc.edu

Section S1. CV Analysis for Different Cu Deposition

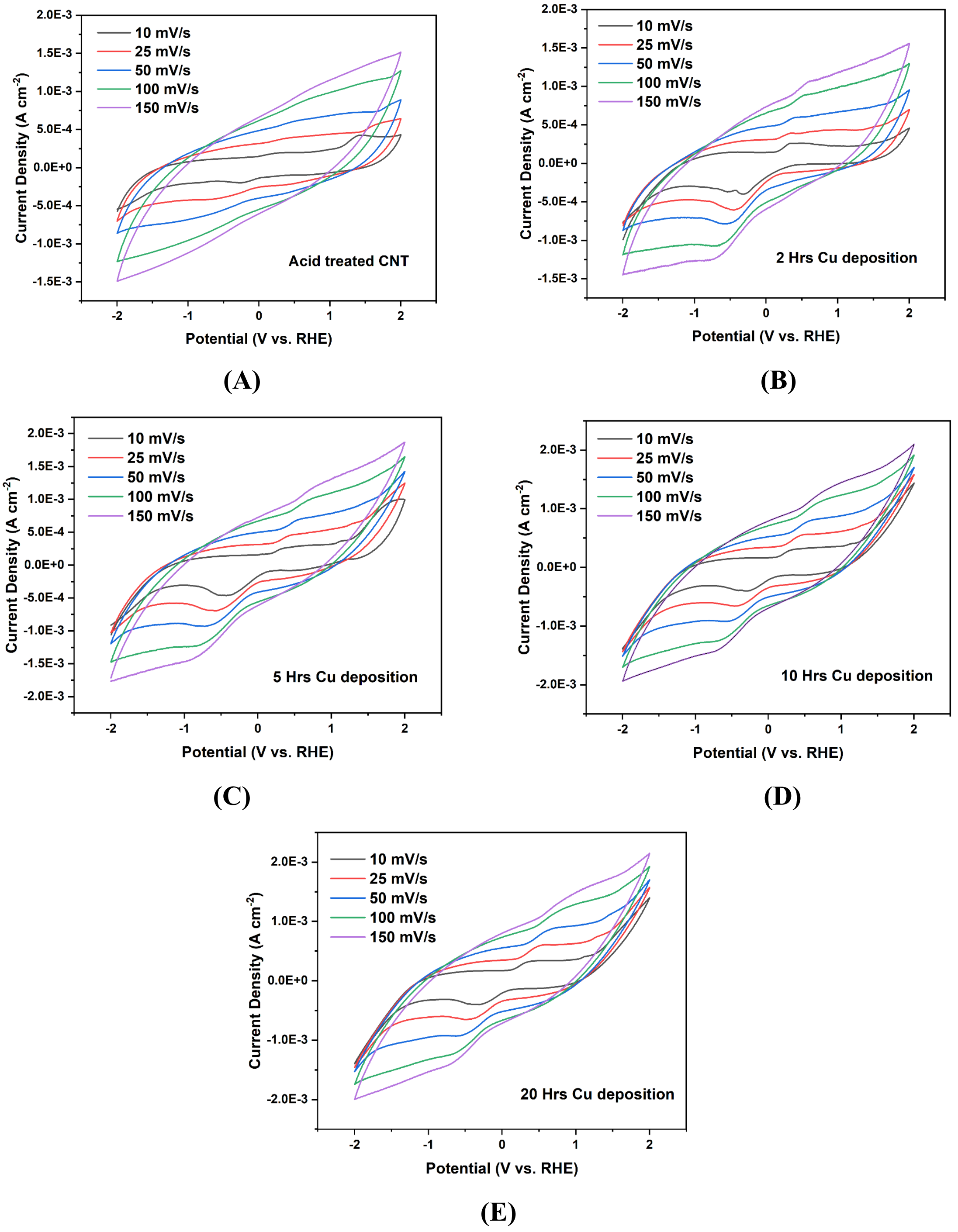

Figure S1. CV graphs of (A) acid treated CNT sheet (no Cu deposition); (B) activated and 2 hours Cu deposition; (C) activated and 5 hours of Cu deposition; (D) activated and 10 hours of Cu deposition; and (E) activated and 20 hours of Cu deposition.

Section S2. Current Density for Different Cu Depositions

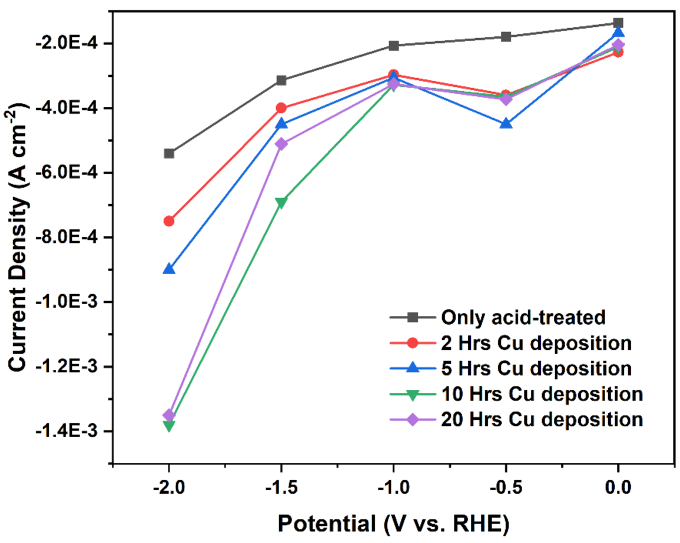

Figure S2. Current density comparison during CO_2 reduction between CNT samples with different Cu deposition.

Section S3. The discharging time graph (using $NaHCO_3$ electrolyte on both sides)

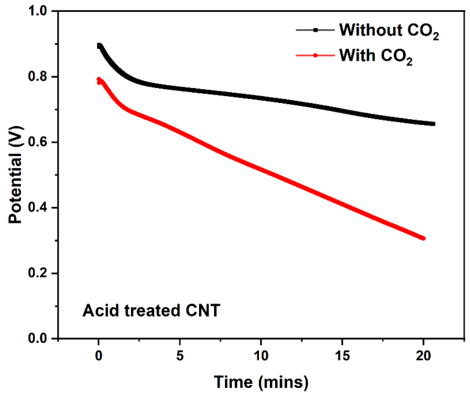

(A)

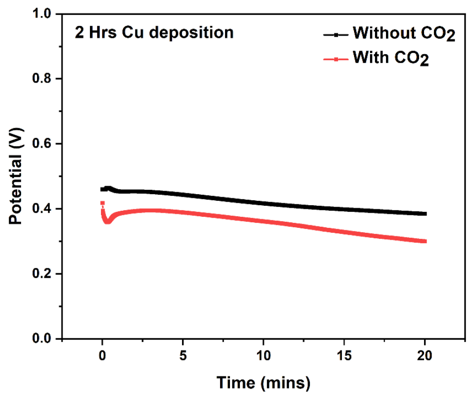

(B)

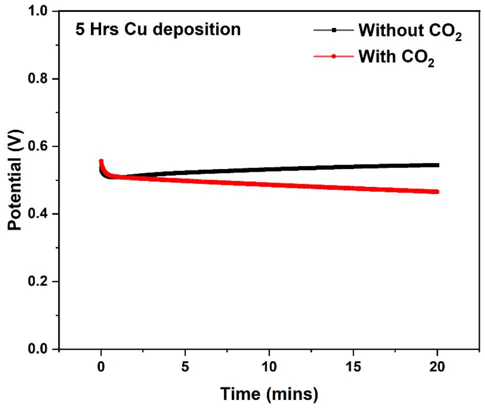

(C)

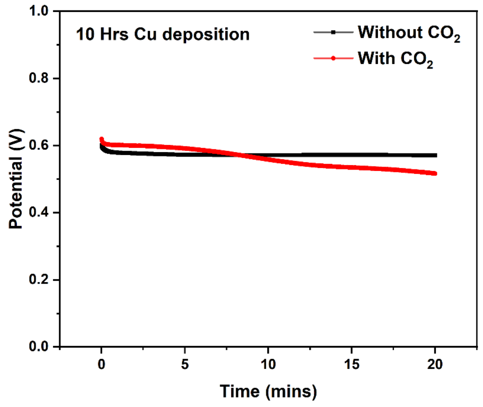

(D)

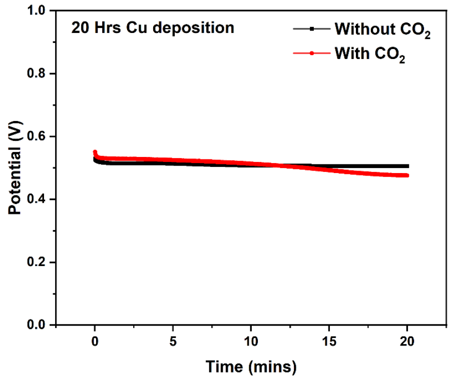

(E)

Figure S3. CV graphs ($NaHCO_3$ electrolyte as the electrolyte) of (A) acid treated CNT sheet (no Cu deposition); (B) activated and 2 hours Cu deposition; (C) activated and 5 hours of Cu deposition; (D) activated and 10 hours of Cu deposition; and (E) activated and 20 hours of Cu deposition**.**

Section S4. Calculation of Theoretical Potential of Zn-CO_2 battery

According to the results and analysis, the following reactions could take place:

Cathode: $2CO_2 + 8H^+ + 8e^{-1} \rightarrow CH_3COOH + 2H_2O$ (1M NaCl, sat. CO_2) **(S4-1)**

$$E_c = E^{\theta}_{CO_2/CH_3COOH} - \frac{RT}{nF}\ln\left[\frac{\alpha_{CH_3COOH}\times\alpha^2_{H_2O}}{\alpha^2_{CO_2}\times\alpha^8_{H^+}}\right] = 0.32\ V - \frac{8.314\times 298.15}{8\times 96485}\ln\frac{0.03}{1\times(10^{-5.8})^8} = -0.039\ V$$

(S4-2)

Anode: $4Zn + 8OH^- - 8e^{-1} \leftrightarrow 4Zn(OH)_2$ (1M KOH)

$$E_a = E^{\theta}_{Zn(OH)_4^{2-}/Zn} - \frac{RT}{nF}\ln\left[\frac{\alpha_{Zn(OH)_4^{2-}}}{\alpha_{Zn}}\right] = -1.199 - \frac{8.314\times 298.15}{2\times 96485}\ln\left[\frac{0.02}{1}\right] = -1.149\ V \quad \textbf{(S4-3)}$$

Overall reaction: $4Zn + 2CO_2 + 8OH^- + 8H^+ \rightarrow 4Zn(OH)_2 + CH_3COOH + 2H_2O$ **(S4-4)**

Therefore, the theoretical electromotive force of Zn-CO_2 battery:

$E_{Theoretical} = E_c - E_a = 1.11\ V$ **(S4-5)**

And theoretical energy density (ED) of the battery is

$ED_{Zn} = C_{Zn} \times E_{theo} = 825\ mAh\ g^{-1} \times 1.11V = 915.75\ Wh\ kg^{-1}$ **(S4-6)**

In above equations, R is 8.314 (molar gas constant), T is 298.15K, n is number of electrons transferred per mole of product, F is 96485 C/mol (Faradaic constant), α is the chemical activity of corresponding ions or molecules, C_{Zn} is the theoretical capacity of the Zn anode-based battery.

Section S5. Table of performance comparison between this work and other published work

Table S1. Table of performance comparison between this work and other published work.

**Cathode**	**Anode**	**Electrolyte**	**Potential**	**Main Byproducts**	**Ref**
Cu deposited CNT	Zinc	NaCl +KOH	1.6	Acetic acid	**This work**
Carbon Hollow fiber	Zinc	[EMIM] [BF4]/ [EMIM] [BF4]	1.01	Methane	[1]
Palladium	Zinc	KOH + NaCl/ H2O	0.955	Formic acid	[2]
CNT@Ni, CNT@Cu	Zinc	[EMIM] [BF4]	0.98, 0.82	CO, H_2	[3]

References

[1] K. Wang, Y. Wu, X. Cao, L. Gu, J. Hu, A Zn–CO_2 Flow Battery Generating Electricity and Methane,Advanced Functional Materials. (2020). https://doi.org/10.1002/adfm.201908965.

[2] J. Xie, X. Wang, J. Lv, Y. Huang, M. Wu, Y. Wang, J. Yao, Reversible Aqueous Zinc–CO 2 Batteries Based on CO_2 –HCOOH Interconversion , Angewandte Chemie. 130 (2018) 17242–17247. https://doi.org/10.1002/ANGE.201811853.

[3] Y. Chen, Y. Mei, M. Li, C. Dang, L. Huang, W. Wu, Y. Wu, X. Yu, K. Wang, L. Gu, L. Liu, X. Cao, Highly selective CO_2 conversion to methane or syngas tuned by CNTs@non-noble-metal cathodes in Zn-CO_2 flow batteries, Green Chemistry. 23 (2021). https://doi.org/10.1039/d1gc02496e.
